# Reading the Wrong Way with the Right Hemisphere

**DOI:** 10.3390/brainsci3031060

**Published:** 2013-07-17

**Authors:** Karen E. Waldie, Charlotte E. Haigh, Gjurgjica Badzakova-Trajkov, Jude Buckley, Ian J. Kirk

**Affiliations:** School of Psychology, The University of Auckland, Private Bag 92019, Auckland 1142, New Zealand; E-Mails: char.haigh@gmail.com (C.E.H.); g.badzakova@auckland.ac.nz (G.B.-T.); j.buckley@auckland.ac.nz (J.B.); i.kirk@auckland.ac.nz (I.J.K.)

**Keywords:** cerebral laterality, dyslexia, fMRI, lexical decision, reading disability, phonological processing

## Abstract

Reading is a complex process, drawing on a variety of brain functions in order to link symbols to words and concepts. The three major brain areas linked to reading and phonological analysis include the left temporoparietal region, the left occipitotemporal region and the inferior frontal gyrus. Decreased activation of the left posterior language system in dyslexia is well documented but there is relatively limited attention given to the role of the right hemisphere. The current study investigated differences in right and left hemisphere activation between individuals with dyslexia and non-impaired readers in lexical decision tasks (regular words, irregular words, pseudowords) during functional Magnetic Resonance Imaging (fMRI). Results revealed the expected hypo-activation in the left posterior areas in those with dyslexia but also areas of overactivation in the right hemisphere. During pseudoword decisions, for example, adults with dyslexia showed more right inferior occipital gyrus activation than controls. In general the increased activation of left-hemisphere language areas found in response to both regular and pseudowords was absent in dyslexics. Laterality indices showed that while controls showed left lateralised activation of the temporal lobe during lexical decision making, dyslexic readers showed right activation. Findings will inform theories of reading and will have implications for the design of reading interventions.

## 1. Introduction

Reading is a complex cognitive task which is acquired relatively slowly throughout childhood and requires explicit teaching and effort. To read and write well, a person needs orthographic knowledge, which involves the ability to recognize the visual form of a word or string of letters in order to translate spoken language into a written form, as well as phonological awareness, which is the ability to understand sound structures and detect phonemes [1,2]. Prominent computational models of reading, such as the parallel distributed processing (PDP) group of models [3,4,5] posit that orthographic and phonological components operate cooperatively to activate lexical semantics. The PDP group of models emphasise both speed and accuracy in the parallel processing of phonological, orthographic and semantic information. Reading is thought to begin primarily as a phonological process, with phonological awareness one of the strongest predictors of reading success [6]. 

Skilled word reading, regardless of language, depends on a left-lateralized network of frontal, temporoparietal, and occipitotemporal areas of the brain. Functional Magnetic Resonance Imaging (fMRI) studies have shown that the temporoparietal cortex is involved in grapheme-phoneme conversion [7]. The occipitotemporal (OT) region is important for visual and orthographic encoding (whole word recognition) and includes the visual word-form area (VWFA [8]). The OT region has a strong reciprocal relationship with the left inferior frontal gyrus (IFG; Broca’s area) [9]. The IFG is associated with articulation and is also involved in phonological processing [10]. Activation in this area is positively correlated with reading ability [10]. Both real words and pseudowords are processed within this left hemisphere neural network [11].

Right hemisphere involvement is also common in beginning reading [12,13]. Children show bilateral activation in the superior and middle frontal areas during reading tasks and right hemisphere activity declines as reading develops [14]. Learning to read is also associated with decreasing reliance on right extrastriate and inferotemporal cortices [10]. In dyslexic children, however, recent research shows that recruitment of right hemisphere frontal regions plays an increasing role over time in reading development [15].

Some children have great difficulty attaining fluent single word reading and, if it persists, may have a specific reading disability (herein called dyslexia). Dyslexia is a persistent and unexplained failure to achieve accurate and/or fluent word recognition skills, despite adequate intelligence, intact senses, and proper instruction [16]. While many adults who struggle to read in childhood are eventually able to read accurately, their reading often remains slow and effortful with persistent spelling and written expression deficits [17]. 

The primary cognitive deficit in dyslexia can be traced back to deficient phonological coding [18,19], which impairs the way that speech sounds are represented, stored and retrieved [16]. Indeed, a disruption in the ability to link graphemes and phonemes in individuals with dyslexia is shown to be related to structural [20,21,22,23,24] and functional [25,26,27] abnormalities. For example, the parietal operculum is less asymmetrical in dyslexics than in controls and the degree of asymmetry is inversely related to phonological task performance [28]. Dyslexic adults also show reduced left but increased right hemisphere activation in temporoparietal regions during phonological processing, a pattern the opposite of that observed in typical readers [29]. 

More recent reviews of fMRI studies have similarly emphasised a reduction or absence of activity in the left hemisphere temporoparietal region (crucial for phonological processing and phoneme-grapheme conversion) and OT region, including the VWFA, in dyslexic individuals during language tasks [30,31,32,33]. A meta-analysis of 17 fMRI studies confirmed underactivation of left temporoparietal and OT areas during dyslexic reading and the authors stress that underactivation is likely to be a cause rather than a consequence of reading impairment [34]. Grapho-phonological processing in the OT rather than in the TP region has also been recently emphasized (e.g., [35]). 

Compared to numerous reports of abnormal left hemisphere networks in dyslexia, activation in the right hemisphere has received relatively little theoretical and empirical attention. There are notable exceptions to this, including studies using Positron Emission Tomography (PET), fMRI, and event-related potentials (ERP). Rumsey and colleagues were the first brain imaging investigators to report a rightward shift in activation in the inferior parietal lobe in adults with dyslexia during language tasks [36]. Pugh *et al.* [37] later found increased functional connectivity between the angular gyrus and related sites in the right hemisphere of dyslexic adults. Dyslexic readers have also shown greater ERP activation of the right hemisphere in processing both words and pseudowords [38]. In an fMRI study examining age-related changes in brain activity during a pseudoword task (*do* “*leat*” *and* “*jete*” *rhyme*?), the left IFG and the left posterior medial OT area became more active with increasing age in children with dyslexia*.* Importantly, compared to typical readers dyslexic children continued to show right hemisphere involvement [13]. In a recent auditory word rhyming fMRI study, dyslexic children showed an over-reliance on right posterior cortex for phonological processing [39]. Such results emphasise that there are lateralized neural differences between dyslexic and typical readers during reading tasks. 

Dyslexic individuals appear to rely on the same neural networks than typical readers; however, they show less activity in left hemisphere networks and an atypical pattern of continued right hemisphere involvement. Activations in right hemisphere regions are more robust in dyslexics compared to typical readers [40]. These findings suggest that atypical activation in the right hemisphere may provide evidence of a neurobiological signature for dyslexia. It is likely that right hemispheric activity, observed in both dyslexic adults and children, is a compensatory mechanism due to the extra cognitive effort and attention demand required during phonological processing [41,42,43,44,45,46]. It is unclear, however, if right hemisphere involvement has etiological importance or whether these areas just take part in compensatory processes. As recently emphasized, fMRI findings on the right hemisphere are scarce and unsystematic [47].

Taken together, researchers have not yet reached a consensus on the neural basis of dyslexia. In addition to the questions remaining regarding right hemisphere participation, there is disagreement among researchers as to the involvement of the left inferior frontal cortex during reading [31]. Whereas some researchers have consistently stressed that over-activation of the left inferior frontal cortex plays a compensatory role in older children with dyslexia [25], others have not found support for this conclusion [30]. The developmental role of the temporoparietal region has also been recently called into question. In a second meta-analysis, left temporoparietal hypoactivation was consistently found only for dyslexic adults but not for dyslexic children [47]. 

Here, our aim is to address questions regarding left and right brain activity in dyslexia by using fMRI during three lexical decision tasks: regular words; irregular words; and pseudowords. It is not known if the right hemisphere is differently activated in dyslexia for all word types. Activity during each task was compared to both a resting (fixation) condition and to age-, gender- and IQ-matched typically reading adults. Temporal and frontal lobe laterality indices were also analysed. We asked the following questions: Does right hemisphere overactivity occur only during phonological processing? Are right brain areas differently involved according to word type? Identifying activation patterns in right brain systems may provide an important means of precise identification and evidence-based interventions for dyslexia. 

## 2. Results

### 2.1. Behavioural Results

Accuracy Scores were subjected to a 2 × 5 Split-Plot Analysis of Variance (ANOVA), with Group (dyslexic and controls) as the between-subjects factor and Task (letters, regular words, irregular words, pseudoword) as the within-subjects factor. There was only a significant effect of task (F_(1.54,20.41)_ = 8.87, *p* = 0.004) whereby the pseudoword task was significantly less accurate overall (M = 87.2%, SD = 2.7) than the regular word task (M = 96.8%, SD = 1.6). 

### 2.2. Functional MRI Results

**Figure 1:**
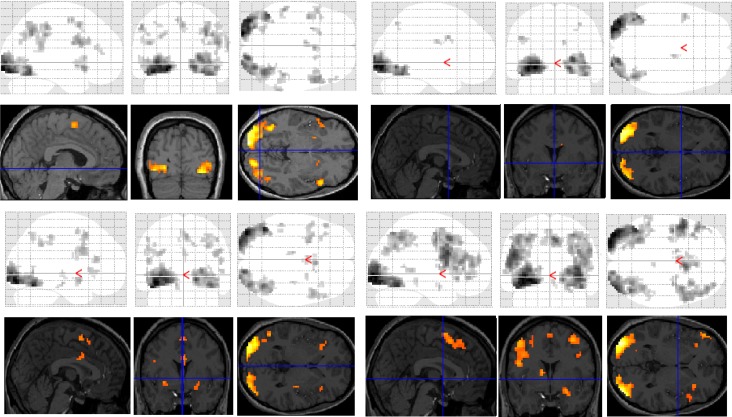
Significant clusters of activation displayed on section overlay and glass brain statistical parametric maps (SPMs) observed in control participants for each task contrast (*versus* baseline fixation): letter case judgment (top left); regular word decision (top right); irregular word decision (bottom left); pseudoword decision (bottom right).

**Figure 2:**
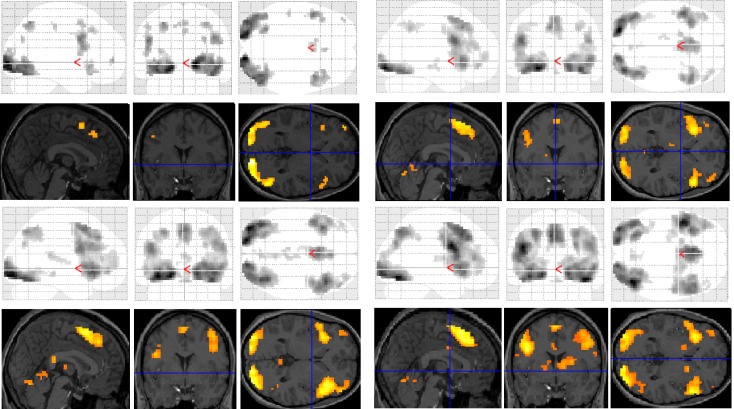
Significant clusters of activation displayed on section overlay and glass brain SPMs observed in participants with dyslexia for each task contrast (*versus* baseline fixation): letter case judgment (top left); regular word decision (top right); irregular word decision (bottom left); pseudoword decision (bottom right).

Functional MRI results for each of the subjects were analysed and organised according to the contrast of interest (*versus* baseline fixation): (1) letter case judgment; (2) regular word decision; (3) irregular word decision; (4) pseudoword decision. Significant clusters of activation for each contrast on section overlay and glass brain statistical parametric maps (SPMs) are illustrated separately for control (Figure 1) and dyslexic (Figure 2) participants. 

**Figure 3:**
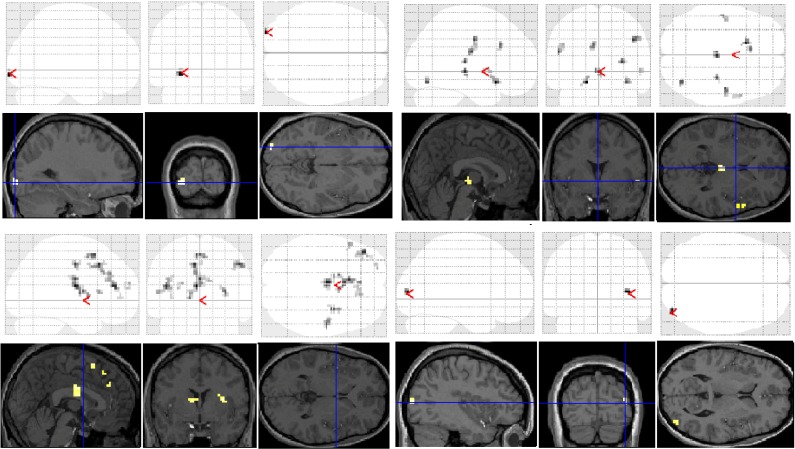
Significant differences between groups for each task contrast displayed on section overlay and glass brain SPMs: regular word decision: Controls > Dyslexics (top left); regular word decision: Dyslexics > Controls (bottom left); pseudoword decision: Dyslexics > Controls (top right); pseudoword decision: Controls > Dyslexics (bottom right).

Significant group differences in brain activation for each of the contrasts are presented in Table 1 and illustrated in Figure 3. There were no significant differences for the letter case judgment and irregular word tasks.

Significant group differences were observed in the regular word comparison, with typical readers showing greater activation than the dyslexic group in the left inferior occipital gyrus. Dyslexic participants showed greater activation than controls in the left pallidum, the middle cingulate cortex, the cingulate and superior medial gyri, and the middle and superior frontal gyri than non-impaired readers. Dyslexic participants also showed significantly greater activation in the right putamen and precentral gyrus compared with non-impaired readers.

For the irregular word comparison the analysis yielded no significant differences in hemispheric activation between the dyslexic and non-impaired reader groups. Dyslexic and typical readers showed significant bilateral activation in the inferior occipital gyri, fusiform gyrus, IFG, and lingual and angular gyri.

For the pseudoword decision comparison, the analysis revealed significant group differences in both right and left hemisphere activity. In the left hemisphere, dyslexic participants showed greater activation in the putamen, the middle cingulate cortex and the rectal and postcentral gyri than non-impaired readers. In the right hemisphere, dyslexic participants showed lower activation in the right middle occipital gyrus than non-impaired readers but greater activation in the precentral and inferior occipital gyrus, the insula lobe, and the rolandic operculum.

**Table 1:** Brain regions showing significant differences between dyslexic and control participants for the contrasts of interest. The number of voxels in significant clusters, with Montreal Neurological Institute (MNI) coordinates for the peak activation voxel, corresponding *t*-values and direction of difference are also shown. More than one local maxima more than 8mm apart are shown where appropriate.

Region	Number of Voxels	*T*	*X Y Z*
**Regular *versus* Fixation**			
*Controls* > *Dyslexics*			
(1) L. Inferior Occipital G.	13	3.99 *	−27 −96 −6
*Dyslexics* > *Controls*			
(1) L. Cingulate G.	64	4.29 *	−3 −9 18
L. Pallidum		3.84 *	−12 6 3
(2) L. SMA	65	4.11 *	−6 21 45
L. Superior Medial G.		3.74 *	−3 36 33
L. Middle Cingulate Cortex		3.67 *	−3 24 36
(3) L. Middle Frontal G.	41	4.10 *	−42 42 36
L. Inferior Frontal G. (Triangularis)		3.55 *	−39 33 24
(4) R. Precentral G.	27	4.04 *	51 −9 45
(5) L. SMA	22	4.00 *	−3 15 57
R. SMA		3.68 *	6 6 54
(6) R. Putamen	22	3.93 *	33 0 12
(7) L. Inferior Frontal G.	10	3.58 *	−51 21 9
(8) L. Superior Frontal G.	10	3.52 *	−21 60 21
L. Middle Frontal G.		3.42 *	−27 57 15
**Pseudoword *versus* Fixation**			
*Controls* > *Dyslexics*			
(1) R. Middle Occipital G.	14	4.27 *	39 −84 9
*Dyslexics* > *Controls*			
(1) L. Rectal G.	13	4.77 *	−15 18 −12
L. Putamen		3.58 *	−12 12 −5
(2) R. Precentral G.	18	4.31 *	45 −9 39
(3) L. Postcentral G.	19	4.29 *	−51 −12 24
(4) R. Insula Lobe	10	4.21 *	30 −21 12
(5) R. Inferior Occipital G.	11	4.11 *	36 −69 −12
(6) L. Middle Cingulate Cortex	13	3.99 *	−6 24 33
(7) R. Rolandic Operculum	14	3.56 *	54 12 0

* *p* < 0.001 uncorrected.

### 2.3. Laterality Index (LI) Analysis

A Group (2) by Task (4) mixed ANOVA was conducted on the LI temporal lobe data. There was a significant main effect of Group (*F*(1, 26) = 4.87, *p* = 0.036) where adults with dyslexia show greater right activation (*M* = −0.014 ± 0.08) and controls show greater overall left activation (*M* = 0.220 ± 0.07). The Group × Condition interaction was not significant, *F*(3, 78) = 1.19, n.s., but the Quadratic trend approached significance (*F*(1, 26) = 2.65, *p* = 0.10). In the pseudoword task, the dyslexic group showed more right hemisphere activation (*M* = −0.08, *SD* = 0.49) and controls showed more left activation (*M* = 0.27, *SD* = 0.34). The findings suggest that dyslexics are more right lateralised in the temporal lobe, particularly when reading pseudowords. 

The same analysis conducted in the frontal lobe revealed no significant main or interaction effects. 

## 3. Discussion

The purpose of this study was to examine differences in right and left hemisphere activation between adults with dyslexia and typical readers in different lexical decision tasks (regular words, irregular words, and pseudowords in separate blocks). Only lexical decisions with regular words and pseudowords showed significant group differences in activation. The accuracy data did not show any group effects. Together the results provide tentative support that the left and right hemispheres play a role in skilled reading and right hemisphere overactivity may be an important marker of dyslexia. 

Consistent with previous reports, participants with dyslexia exhibited significant underactivation of the left OT cortex, including the inferior occipital gyrus, during the regular word lexical decision task (*is BANK a real word or not*?). The left inferior occipital gyrus and the posterior parts of the fusiform and inferior temporal gyrus are involved in the integration of visual elements into perceptual wholes (single objects) [48]. The VWFA in particular is thought to reflect increasing expertise with orthography-phonology connections [7]. 

Group differences were not significant during irregular word lexical decisions (*is SHOE a real word or not*?) with both groups showing similar bilateral frontal and posterior network activation. Irregular words are common in the English language. They must be memorized as they do not conform to the regular grapheme-phoneme conversion rules. As deficient phonological coding is a key cognitive marker of dyslexia, these individuals typically compensate by having strong sight vocabularies. As such, it is perhaps not surprising there were no group differences for this task. 

During pseudoword decision making (*does PHEAT sound like a real word or not*?) dyslexics showed overactivation in multiple right hemisphere regions, including the precentral and inferior occipital gyri, the insula lobe, and the rolandic operculim. Dyslexic children similarly show an over-reliance on right posterior regions for phonological processing [29,39]. Involvement of right hemisphere posterior regions by dyslexics most likely indicates reliance on visuo-perceptual strategies for word recognition [49]. As a compensatory strategy, however, relying on the right hemisphere to read pseudowords is likely to fail, as the right hemisphere is not specialised for grapheme-phoneme tasks. The control group showed the typical left lateralized activation of the language network when reading pseudowords. 

Our laterality index analysis also revealed slightly more right- than left-temporal activity. Laterality indices range in values from −1 to +1, with the extremes representing right hemispheric and left hemispheric lateralisation, respectively. Our adults with dyslexia showed slightly greater right- than left-activation (mean of −0.014) whereas the controls showed overall left activation (mean of 0.22). We therefore observed a lack of the typical left-lateralized activation during decision making tasks in the dyslexic adults.

Laterality indices showed no groups differences in frontal activation. The role of more anterior parts of the language area, namely posterior frontal regions, is less clearly defined in the reading literature. Although Broca’s area has been shown to be active during subvocal rehearsal of phonological material, its pattern of activation in dyslexia has been variably found as inactive [50], normal [30] and overactive [14,51].

Our participants with dyslexia also showed overactivation in the right hemisphere regions of the putamen and precentral gyrus during both regular and pseudoword lexical decision making. The putamen comprises part of both the dorsal striatum and the basal ganglia and is connected to the substantia nigra and globus pallidus. Though the putamen is thought to have no specific specialization, it is involved in regulating movements and implicit learning [52]. The precentral gyrus is also associated with initiating the onset of movements. It is likely that this activity reflects increased reliance on silent articulatory processes. Other studies are consistent with this finding with dyslexic overactivation in precentral/motor regions during phonological reading tasks [53].

Dyslexics also showed greater activation than controls in four left hemisphere regions during both regular and pseudoword lexical decision making (the cingulate and superior medial gyri, middle and superior frontal gyri). These regions are associated with attentional processes and these findings might reflect the engagement of phasic attention as participants detected covertly phonetic changes in stimuli. Interference of the background noise produced by the scanner might have also contributed to this effect, as right hemisphere activity has been associated with perceptually difficult auditory tasks in typical readers [54]. The greater activation of the middle and superior frontal gyri supports reports that left frontal language regions exhibit overactivation in dyslexic readers in order to compensate for the dysfunction in posterior language regions [53]. 

There are important limitations of this study and therefore caution should be used when interpreting the findings. Firstly we were unable to collect response time data in our scanner because of technical issues. We stressed to participants the importance of accuracy over speed and this is perhaps reflected in our behavioural data. Secondly we used uncorrected *p* values to present our BOLD findings and group differences. We did not have enough trials per condition to use corrected values. It was partially an ethical decision based on the fact that our sample was not comfortable spending more time in the scanner performing reading tasks. The lack of statistical power is a serious limitation and it would have been preferable to include more participants. Finally, we did not investigate the occipital lobe laterality index data. Future studies should examine these potential laterality differences and provide more fine-grained analyses rather than whole-lobe differences. 

## 4. Experimental Procedure

### 4.1. Participants

Twelve adults with dyslexia (8 male, 4 female; Mean age 31 ± 9.4 years) and 16 controls (10 male, 6 female; Mean age 30 ± 7.2 years) were included in the final sample. 

Participants were recruited through student learning centers, advertisements and media publicity. All volunteers reported persistent and severe reading difficulties since primary school (dyslexia), or no history of learning difficulties (controls) and were all right-handed, as determined with the Edinburgh-Inventory [55]. Groups were well balanced for education and socio-economic status. 

Adults with dyslexia and control participants were interviewed and were administered a battery of tests by a neuropsychologist that included the Wechsler Adult Intelligence Scale [56], a standardized online reading test [57], and the Woodcock-Johnson Tests of Achievement [58]. Inclusion criteria for individuals with dyslexia included reading performance at least 2 standard deviations below the population mean on the Word-Attack and Word identification subscales of the Woodcock-Johnson, and scores in the reading-impaired range according to the Coltheart criteria. A screener for ADHD (Adult Self Report Scale) was also used to rule out ADHD. Participants were excluded if they had a history of neurological disorder (other than depression or anxiety), major head injury, English as a second language, non-standard schooling, vision/hearing impairment, or IQ < 85. As shown in Table 2, there were no significant differences in IQ or handedness between the experimental groups.

**Table 2:** Sample characteristicswith standard error in parentheses.

	Control	Dyslexia
*n*	16	12
Right handed	84%	83%
Education, years from age 6	15.0 (0.3)	15.6 (0.6)
Parents education, yrs from age 6	13.2 (0.4)	13.8 (0.4)
WJ word ID standard score	108 (2)	88 (2) *
WJ word attack standard score	112 (2)	91 (2) *
WRAT spelling standard score	113 (2)	88 (2) *
WASI IQ—full	120 (1)	120 (2)
WASI IQ—verbal	121 (2)	117 (3)
WASI IQ—performance	114 (2)	118 (2)

* Significant *p* < 0.001, independent *t*-test.

### 4.2. Stimuli and Procedure

A blocked experimental design with a “go/no-go” lexical decision response paradigm was employed in this study (e.g., participants were instructed to respond to a correct answer with their right-hand by pressing a mouse button and refrain from responding for the incorrect response). Ten experimental and ten fixation/baseline blocks were used for the four conditions/tasks in the study (two experimental blocks per condition). Each experimental block lasted for 48 s and was preceded by an 18 s fixation/baseline block. Stimuli were presented in Courier New Bold, 35 font size, using E-Prime [59]. Twenty stimuli were randomly presented in a black font on a grey background for each experimental block. Each stimulus was presented for 2000 ms followed by a 400ms interstimulus interval, which was a blank grey screen.

The lettercase judgment task consisted of upper-case and lower-case letters (e.g., NKWZL or jdfgn). The participant was required to press the mouse button for upper-case stimuli (50% of trials). The regular word lexical decision task consisted of real words (concrete nouns) and pronounceable pseudowords matched on length (e.g., BANK or LORC). The participant was required to press the mouse button for stimuli that were real words (50% of trials). Stimuli in the irregular word condition were words which did not follow the grapheme-phoneme correspondence rules, and pronounceable pseudowords matched on length (e.g., SHOE or NINT). The participant was required to press the mouse button for stimuli that were real words (50% of trials). 

Stimuli in the pseudoword lexical decision task (also referred to as the pseudoword decision task) were all pseudowords [11], but half were pseudohomophones (e.g., BRANE), and pronounceable nonsense words again matched on length (e.g., BRONE). The participant was required to press the mouse button for stimuli that sounded like a real word. Subjects were instructed to think but not speak out loud. 

All participants were required to run through as many practice trials of the experimental block as necessary to achieve 90% accuracy. No feedback was given during the experimental trials. The accuracy data were also recorded. All procedures were approved by the University of Auckland Human Participants Ethics Committee. 

### 4.3. Image Acquisition

Images were acquired using a 1.5T Siemens Avanto scanner (Erlangen, Germany). Scanning sessions began with acquisition of T1-weighted structural volumes using 3D MP-RAGE sequence (TR = 11 ms; TE = 4.94 ms; flip angle: 15°; FOV: 25.6 × 20.8 cm; 170 to 176 axial slices parallel to the AC-PC line; matrix size: 256 × 208; interslice gap: 0 mm; resulting in 1 × 1 × 1 mm voxels, axial acquisition, parallel to AC-PC line, ensuring whole brain coverage). 32 scans were acquired for each condition along with 60 scans for the fixation period resulting in a total of 220 T2*-weighted volumes for each of the subjects (nonverbal, lettercase judgment, regular, irregular, and pseudoword task) in the EPI sequence. In addition, 2 initial “dummy” scans were also collected at the beginning of each sequence to control for T1 saturation but these were not included in the analysis. 

The EPI acquisition sequence parameters were as follows: TR = 3000 ms; TE = 50 ms; flip angle = 90°; FOV = 19.2 cm; matrix size: 64 × 64; with interleaved slice acquisition, starting at the bottom; 30 slices parallel to AC-PC line; slice thickness: 4 mm; 25% gap: resulting in 3 × 3 × 5 mm voxels; whole brain coverage of 150 mm.

### 4.4. Image Pre-Processing and Analysis

SPM5 software (Wellcome Department of Imaging Neuroscience, London, UK) was used for image processing and analysis. The first volume of the first session was used as a reference for coregistration of the first volume for the rest of the sessions. The remaining volumes were realigned to the first volume within each session and a mean of all volumes across the conditions were created. 

The T1-weighted structural image was coregistered to the mean of the functional volumes. By using the unified segmentation procedure, normalisation parameters were estimated. This was then used to normalise both the functional and structural images to the stereotactic coordinate system defined by the Montreal Neurological Institute. Lastly, the functional volumes were then spatially smoothed using an anisotropic Gaussian filter of 9 9 15 mm (three times the voxel size) at full-width at half-maximum (FWHM). 

For each participant, the pre-processed functional volumes were subjected to 1st-level or fixed-effects analysis using the general linear model applied at each voxel across the whole brain. Conditions were modelled by boxcar waveform convolved with a canonical haemodynamic response function. Contrast images of interest were also produced for: (1) Nonverbal minus (*i.e.*, *versus*) Baseline; (2) Lettercase judgment *versus* Baseline; (3) Regular word *versus* Baseline; (4) Irregular word *versus* Baseline; (5) Pseudoword task *versus* baseline; (6) Pseudoword task *versus* Lettercase judgment. An uncorrected threshold of *p* < 0.001 and a contiguity threshold of 10 voxels was used for each comparison and each subject.

### 4.5. Laterality Index Calculations

A laterality index (LI) was calculated to determine the language lateralisation for each of the subjects for the conditions of interest. This was done by computing the number of voxels that were significantly activated in a region of interest in each cerebral hemisphere using the LI toolbox available from the official SPM website. Laterality indices range in values from −1 to +1, with the extremes representing right hemispheric and left hemispheric lateralisation, respectively.


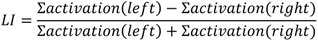


The toolbox applies a bootstrapping technique that allows about 10,000 indices to be calculated at different thresholds yielding a robust mean, maximum, and minimum LI. Taking thresholds into account, an overall weighted bootstrapped LI is calculated. This weighted mean LI was calculated for two regions of interest (ROIs) defined anatomically, the frontal and temporal lobes. These ROIs were predefined in the LI toolbox. 

All statistical analyses were conducted on SPSS 17.0 for Windows and used an alpha level of 0.05 (Greenhouse-Geisser). *Post hoc t*-tests were alpha-adjusted (Bonferroni).

## 5. Conclusions

Our results show that adults with dyslexia are slightly right lateralized overall for language, a profile that differed significantly from the left-lateralized activation observed in typical readers. Though there was also left hemisphere activation observed during reading tasks in the dyslexic participants, the right hemisphere activity was more diverse and primarily occurring in OT regions during pseudoword reading. Right hemisphere compensation in dyslexia may increase as phonological demands increase. Our findings are consistent with earlier work with dyslexic children, suggesting that the activation in the right hemisphere is likely to be a cause rather than a consequence of reading impairment.

Right hemisphere findings should be given more consideration in the literature, particularly as they may have important implications for early intervention, reading remediation and theories of neural plasticity. In 2003 Elise Temple and colleagues showed that auditory processing and oral language training can activate the left posterior reading network in reading disabled children but produces additional compensatory activation in other brain regions [60]. Our findings tentatively support the possibility that right OT compensation might also respond to intensive phonics/phonological processing training. Future designs would need to correlate behavioural measures of reading fluency/accuracy with the right compensatory activity throughout the remediation process to determine how best to accomplish this and whether the right hemisphere participation is helping or hindering the remediation. Such calculations might also address the possibility that the right hemisphere activity is inhibitory rather than compensatory as traditionally assumed. It is still an open question whether right hemisphere activation acts in a compensatory or inhibitory role during single word reading in impaired readers. 

Taken together, in addition to an impaired left hemisphere posterior network, right posterior overactivity may be an important biological marker of dyslexia if our results are replicated. Dyslexic adults appear to compensate for their reading impairment by an increased recruitment of these areas to assist with visual coding. The possibility that the right hemisphere neural mechanisms are inhibitory rather than compensatory should be investigated in further studies.
